# Imported Schistosomiasis: A New Public Health Challenge for China

**DOI:** 10.3389/fmed.2020.553487

**Published:** 2020-10-22

**Authors:** Lei Wang, Xiaoying Wu, Xiaoli Li, Xiaoyan Zheng, Fei Wang, Zhiqun Qi, Minjun Huang, Yang Zou

**Affiliations:** ^1^Emergency and Critical Care Center, Beijing Friendship Hospital, Capital Medical University, Beijing, China; ^2^Beijing Institute of Tropical Medicine, Beijing, China; ^3^Beijing Key Laboratory for Research on Prevention and Treatment of Tropical Diseases, Beijing, China; ^4^Department of Gastroenterology, 3rd Affiliated Hospital, Sun Yat-sen University, Guangzhou, China

**Keywords:** imported schistosomiasis, *Schistosoma haematobium*, *Schistosoma mansoni*, *Biomphalaria straminea*, clinical features

## Abstract

Significantly increased imported schistosomiasis cases have been reported in China as the economy grows. The aim of this study is to review and summarize the current status, clinical features, and transmission risk of imported infections of *Schistosoma mansoni* and *Schistosoma haematobium* in China. A retrospective study was performed to review all information regarding the imported cases of schistosomiasis collected from published literature and the database of the National Notifiable Disease Report System from 1979 to 2019. The characterization of epidemiological and clinical features was analyzed. A total of 355 cases of imported schistosomiasis have been reported in 15 provinces (autonomous regions, municipalities) in China since 1979, including 78 cases of infection with *S. mansoni* (21.97%), 262 cases with *S. haematobium* (73.80%), and 15 cases with unidentified *Schistosoma* species. Eosinophilia was the most common sign of the infection with *S. mansoni* (91.03%). The parasitological findings were confirmed in 89.74% (70/78) of cases infected with *S. mansoni* and 32.06% (84/262) of cases infected with *S. haematobium*. There was no imported case of infection of *Schistosoma japonicum, Schistosoma intercalatum*, or *Schistosoma mekongi* reported in China during this period. Praziquantel is the best therapeutic drug for curing imported schistosomiasis. In addition, *Biomphalaria straminea*, the intermediate host of *S. mansoni*, has already been found in Guangdong province in south China. There is a rising risk that the existence of the intermediate host *B. straminea* and the imported cases of *S. mansoni* infection could cause the spread of the infections and make these endemic. Thus, better understanding of the clinical features and the transmission pattern of these *Schistosoma* infections would assist Chinese physicians in the diagnosis and treatment of these imported schistosomiasis cases.

## Introduction

Schistosomiasis is prevalent in 76 tropical and subtropical countries across the world. Currently, over 700 million people live in endemic areas globally, and more than 240 million people are estimated to be infected, over 90% of them living in Africa (1). There are two main forms of human schistosomiasis: intestinal schistosomiasis and urogenital schistosomiasis; the former is caused by the infections of *Schistosoma japonicum, Schistosoma mansoni, Schistosoma mekongi* and *Schistosoma intercalatum* and the latter is caused by the infections of *Schistosoma haematobium* (2) (Table 1).

**Table 1:** Global geographic distribution of schistosomiasis.

**Type of schistosomiasis**	***Schistosoma* species**	**Geographic distribution**
Intestinal schistosomiasis	*S. mansoni*	Sub-Saharan Africa, the Middle East, the Caribbean, Brazil, Venezuela, and Suriname
	*S. japonicum*	China, Indonesia, and the Philippines
	*S. mekongi*	Several districts of Cambodia and Laos
	*S. intercalatum*	Rainforest areas of central Africa
Urogenital schistosomiasis	*S. haematobium*	Africa, the Middle East, and Corsica (France)

*S. japonicum* is the only species that infects people in China, causing serious schistosomiasis japonicum characterized by liver fibrosis, cirrhosis, and portal hypertension (3). There are no other *Schistosoma* species found in China (4, 5). With the economic boom and increased trade with other countries around the world, China has deepened its economic activities with foreign countries in the fields of infrastructure construction, energy, travel, and trade. There is a tremendously increased number of people from around the world who travel to China for business, academic studies, and tourism. Meanwhile, more and more Chinese people go abroad for economic activities, study, or travel as well (5). The increased international travel and trade tie inevitably imports some diseases that only happen abroad back to China, including schistosomiasis. Based on the database of the National Notifiable Disease Report System (NNDRS), there had been 355 cases of imported schistosomiasis cases reported in 15 provinces (autonomous region, municipalities) in China from 1979 to 2019, including 78 cases infected with *S. mansoni*, 262 cases with *S. haematobia*, and 15 cases with unidentified *Schistosoma* species (5). Due to the existence of the snail *Biomphalaria straminea*, an intermediate host of *S. mansoni*, being already found in Guangdong province in south China, the imported *S. mansoni* increases the risk of its transmission in China (6). To better understand imported schistosomiasis for better diagnosis and treatment and to get prepared for their possible transmission in China, we reviewed and analyzed the clinical features and the characterization of imported schistosomiasis since 1979, including 78 cases of imported schistosomiasis mansoni and 262 cases of schistosomiasis haematobia.

## Materials and Methods

### Database and Literature

All data for those diagnosed with imported schistosomiasis, including parasite species, infection source, and demographic, epidemiological, and clinical information, were collected from published literature and the database of the NNDRS from 1979 to 2019.

### Diagnosis

A definitive diagnosis of schistosomiasis mansoni or haematobia was based on the combination of specific clinical manifestations, travel history in endemic areas or exposure to contaminated water, imaging features of CT or MRI scan, colonoscopy/cystoscopy, egg identified under a microscope in fecal or urine specimens or in biopsy tissues or miracidia hatched from stool samples, and eosinophilia and serological antibody detection with ELISA.

### Ethics

Data were collected from peer-reviewed published literature and public database with approved ethics statement or patients' consent, without exposing the patients' identity information.

## Results

### Characterization of Imported Schistosomiasis Mansoni in China

A total of 78 cases of imported schistosomiasis mansoni were reported in China during the period from 1979 to 2019. These cases were identified in Beijing, Zhejiang, Jiangxi, and Hunan provinces. All cases were Chinese laborers who had working and living experience in African countries, including Ethiopia, Nigeria, Uganda, Zimbabwe, and the Democratic Republic of the Congo, and had a history contacting with contaminated water during daily life activities (Table 2).

**Table 2:** General information of imported schistosomiasis mansoni in China from 1979 to 2019.

**Reported year**	**Reported cites**	**Number of cases**	**Infection source**	**Pattern of infection**	**Time from exposure to diagnosis (months)**	**References**
1979	Beijing	67	Africa	Water contact due to daily life activities, swimming	12–24	(7, 8)
2011	Beijing	2	Ethiopia	Swimming	2	(9)
2014	Zhejiang	1	Nigeria	Contacting with contaminated water twice a day	1.5	(10)
2017	Beijing	6	Ethiopia, Nigeria, Uganda, and Democratic Republic of the Congo	Fishing or swimming in local waters	3	(11)
2017	Jiangxi	1	Zimbabwe	Mining	3	(12)
2019	Hunan	1	Africa	History of multiple swimming activities	2	(13)

**Figure 1:**
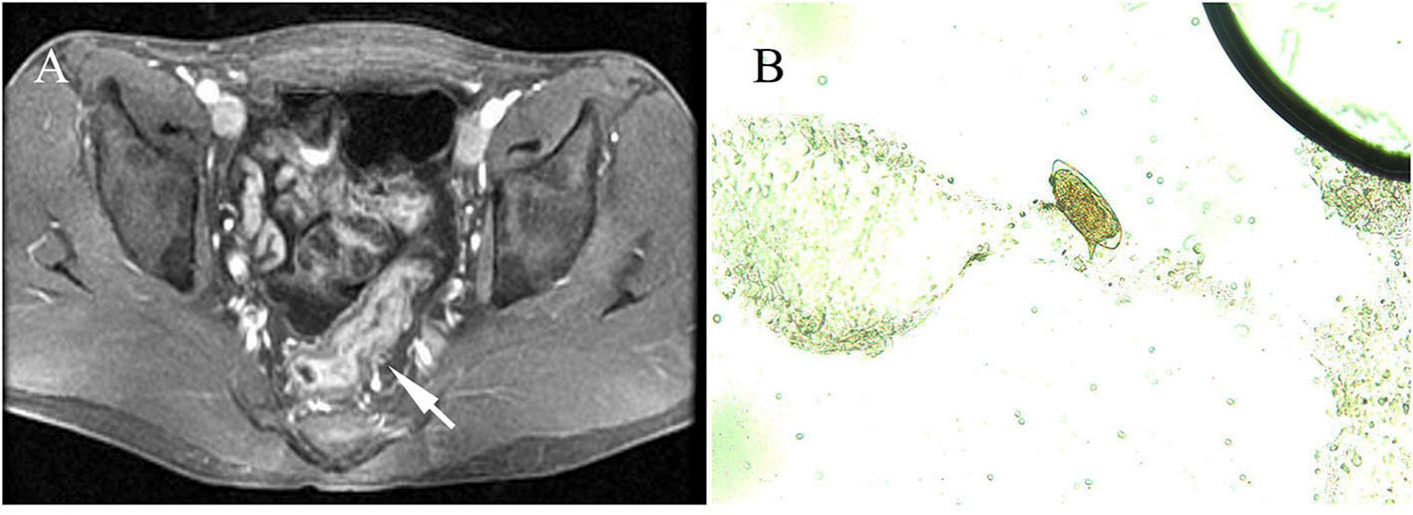
Radiological features and parasitological identification of patients with infections of *Schistosoma mansoni*. **(A)** Wall thickening of the rectum and the sigmoid colon is shown by pelvic MRI scan (pointed arrow). **(B)** *S. mansoni* egg was identified under a microscope (×400) in colon tissue through a biopsy from an infected patient.

The duration from exposure to cercaria-contaminated water to definitive diagnosis was 1.5–24 months; 14.1% (11/78) of the cases had a definitive diagnosis within 3 months from exposure and 87.18% (68/78) for more than 3 months. The main clinical manifestations included weakness (66.67%, 52/78), fever (58.97%, 46/78), diarrhea (55.13%, 43/78), abdominal pain (38.46%, 30/78), mucous and bloody stool (24.36%, 19/78), and sweating (19.2%, 15/78). Three of them had no any symptom (3.85%, 3/78). Physical examinations showed hepatomegaly (57.69%, 45/78), splenomegaly with moderate stiffness (12.82%, 10/78), overreaction of the nervous system (2.56%, 2/78), and rash (1.28%, 1/78). A complete blood count showed that 91.03% (71/78) of the cases had eosinophilia. A total of 2.56% were positive for fecal occult blood test. One case displayed diffused miliary nodule shadows in both lungs, hepatic cirrhosis, splenomegaly by imaging examination (1.28%, 1/78), and thickening of the sigmoid colon and rectum wall as revealed by colonoscopy (1.28%, 1/78) (Figure 1A). Microscopic examination identified *S. mansoni* eggs in their stool samples or miracidia hatched from their stool samples in 69 out of 78 cases (88.46%). Rectal mucosal biopsy revealed *S. mansoni* eggs in 14.10% (11/78) of the cases (Figure 1B; Table 3).

**Table 3:** Clinical features of 78 imported cases of schistosomiasis mansoni in China from 1979 to 2019.

**Features**	**Symptoms and signs**	**Number of positive cases**	**Percentage (%)**
Clinical manifestation	Weakness	52	66.67
	Fever	46	58.97
	Diarrhea	43	55.12
	Abdominal pain	30	38.46
	Mucous and bloody stool	19	24.36
	Sweating	15	19.23
	No symptoms	3	3.85
Physical examination	Hepatomegaly	45	57.69
	Splenomegaly with moderate stiffness	10	12.82
	Symptoms of the nervous system	2	2.56
	Rash	1	1.28
Laboratory tests	Eosinophilia	71	91.03
	Positive fecal occult blood test	2	2.56
Imaging features	Diffuse miliary nodule shadows in both lungs, hepatic cirrhosis, splenomegaly, sigmoid colon and rectum wall thickening	1	1.28
Definitive diagnosis	Identification of *Schistosoma mansoni* eggs in stool samples or miracidia hatched from stool samples	69	88.46
	Detection of *Schistosoma mansoni* eggs using rectal mucosal biopsy	11	14.10

The central nervous system had the most serious ectopic presentation of *S. mansoni* infection. Apparently, both brain and spinal cord can be involved, although it was very rare in clinical practice. Currently, in China, there was one case of imported schistosomiasis mansoni that presented lower extremity numbness and weakness complicated by dysphoria, enlargement of the spinal cord at vertebrae T8 to L2 complicated by abnormal reactions, and intensified foci on plain and contrast-enhanced scans of the spinal cord at the thoracolumbar segment. Antibody testing against *S. japonicum* antigen was cross-positive with titer 1:16, and *S. mansoni* DNA was detected in the cerebrospinal fluid of the patient. Definitively, the case was diagnosed with the identification of typical *S. mansoni* eggs in the feces. The patient received chemotherapy with praziquantel and steroids, which swiftly ameliorated the symptoms and cured the *S. mansoni* infection (13).

### Clinical Analysis of Imported Schistosomiasis Haematobia in China

A total of 262 cases with infection of *S. haematobia* were reported in China from 1979 to 2019 (12, 14–33). All of these cases were imported from abroad and reported in Beijing, Shaanxi, Hubei, Jilin, Fujian, Jiangsu, Hunan, Henan, Guangxi, Shandong, Zhejiang, and Jiangxi provinces (municipality, autonomous region). Among them, 84.73% (222/262) were Chinese who had working experience in 19 African countries including Angola, Mozambique, South Africa, and Nigeria before they returned to China. The most common job involved abroad was construction worker in building railways, airports, and roads (90.99%, 202/222). The other 40 patients were Africans diagnosed with an infection of *S. haematobia*, including 35 male and five females, with a mean age of 20.95 ± 5.49 years. These patients originated from Tanzania (10 cases), Zambia (five cases), Yemen (two cases), Mali (22 cases), and Mozambique (one case) (Table 4).

**Table 4:** General information on 262 imported cases of schistosomiasis haematobia in China from 1979 to 2019.

**Reported year**	**Reported regions**	**Number of cases**	**Country of origin**	**Nationality**	**Time from exposure to diagnosis (months)**	**References**
1980	Beijing	15	Tanzania, Zambia	Foreign	Unknown	(14)
1984	Shaanxi	2	North Yemen	Foreign	Unknown	(15)
1988	Beijing	22	Mali	Foreign	Unknown	(16)
1991	Hubei	1	Egypt	Chinese	9	(17)
1992	Jilin	1	Nepal	Chinese	24	(20)
1992	Beijing	2	Zimbabwe	Chinese	Unknown	(18)
1992	Fujian	21	Yemen	Chinese	Unknown	(19)
2005	Jiangsu	1	Mozambique	Foreign	6	(21)
2007	Shaanxi	1	Angola	Chinese	2	(22)
2011	Hunan	184	Angola, Mozambique	Chinese	Unknown	(23)
2013	Henan	2	Tanzania, Angola	Chinese	1, 3	(25)
2013	Fujian	1	Ghana	Chinese	19	(24)
2014	Hubei	1	Angola	Chinese	7	(26)
2015	Guangxi	1	Angola	Chinese	4	(28)
2015	Shandong	1	Angola	Chinese	12	(27)
2016	Zhejiang	1	Nigeria	Chinese	5	(29)
2017	Fujian	1	Angola	Chinese	7	(30)
2017	Zhejiang	1	Nigeria	Chinese	1	(12)
2017	Jiangxi	1	Zambia	Chinese	12	(33)
2018	Henan	1	Tanzania	Chinese	84	(31)
2019	Jiangsu	1	Sudan	Chinese	14	(32)

**Figure 2:**
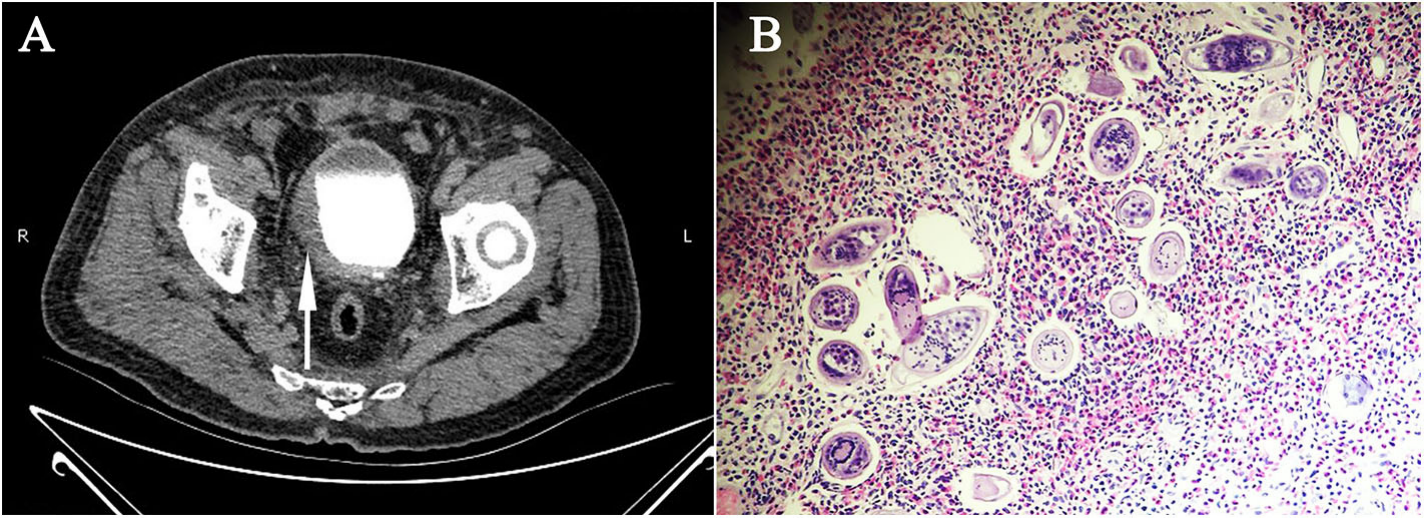
Radiological features and parasitological identification of patients with infections of *Schistosoma haematobium*. **(A)** CT urography showing the thickened and stiff wall of the bladder (pointed arrow) of a patient with chronic infection of *S. haematobium*. **(B)** Vesical biopsy tissue section showing schistosomal eosinophilic granuloma, with *S. haematobium* eggs observed (×400).

The main clinical manifestations of imported *S. haematobia* infection included fever (9.16%, 24/262), painless hematuria (53.81%, 141/262), painful hematuria (11.83%, 31/262), lower abdominal pain (6.48%, 17/262), irritated and frequent urination (11.45%, 30/262), urodynia (7.63%, 20/262), dysuria (2.29%, 6/262), weakness (20.99%, 55/262), and skin rash (8.01%, 21/262). About 27.48% (72/262) of the infected people had no any symptom. A few patients showed tenderness/pain in the bladder region of the lower abdomen (6.48%, 17/262), nodular enlargements, and tenderness/pain in the bilateral spermatic cord and epididymis (0.38%, 1/262), and hepatosplenomegaly (0.38%, 1/262) during physical examination. Laboratory tests showed eosinophilia (13.74%, 36/262), proteinuria, and increased red and white blood cell counts in urine (46.18%, 121/262). Serological antibody test using indirect hemagglutination assay (IHA) and ELISA showed that 72.13% (189/262) of the patients were positive when *S. japonicum* extract antigen was used. Parasitological examination identified the eggs in the urine of 71 out of 262 patients (27.09%) or in the tissues as detected by both cystoscopy (4.20%, 11/262) and rectal biopsy (0.76%, 2/262) (Figure 2; Table 5) (12, 14–33).

**Table 5:** Clinical features of 262 imported cases of *S. haematobium* infection in China from 1979 to 2019.

**Features**	**Symptoms and signs**	**Number of positive cases**	**Percentage (%)**
Clinical manifestations	Painless hematuria	141	53.81
	Pain hematuria	31	11.83
	Lower abdominal pain	17	6.48
	Urgent urination, frequent urination	30	11.45
	Urodynia	20	7.63
	Dysuria	6	2.29
	Fever	24	9.16
	Weakness	55	20.99
	Rash	21	8.01
	No symptoms	72	27.48
Physical examination	Two to three nodular enlargements and tenderness/pain in bilateral spermatic cord and epididymis	1	0.38
	Tenderness/pain in the bladder region of lower abdomen	17	6.48
	Hepatosplenomegaly	1	0.38

It is very common that *S. haematobium* infection was primarily misdiagnosed as urinary tract infection, prostatitis, urinary stone, acute or chronic appendicitis, urinary tuberculosis, enterospasm, and gastrointestinal dysfunction if the patients' travel experience to endemic countries has not been considered and their urine samples have not been carefully examined. Unfortunately, some of the cases were even misdiagnosed as bladder tumor and received unnecessary surgical procedures.

### Imported Schistosomiasis With Unidentified *Schistosoma* Species

There are 15 other cases of imported schistosomiasis reported, with infection of unidentified *Schistosoma* species due to non-specific clinical manifestation, non-specific infection source, and/or no egg of specific species of *Schistosoma* also detected.

### Geographical Distribution of Imported *S. mansoni* and *S. haematobium* Infections in China

**Figure 3:**
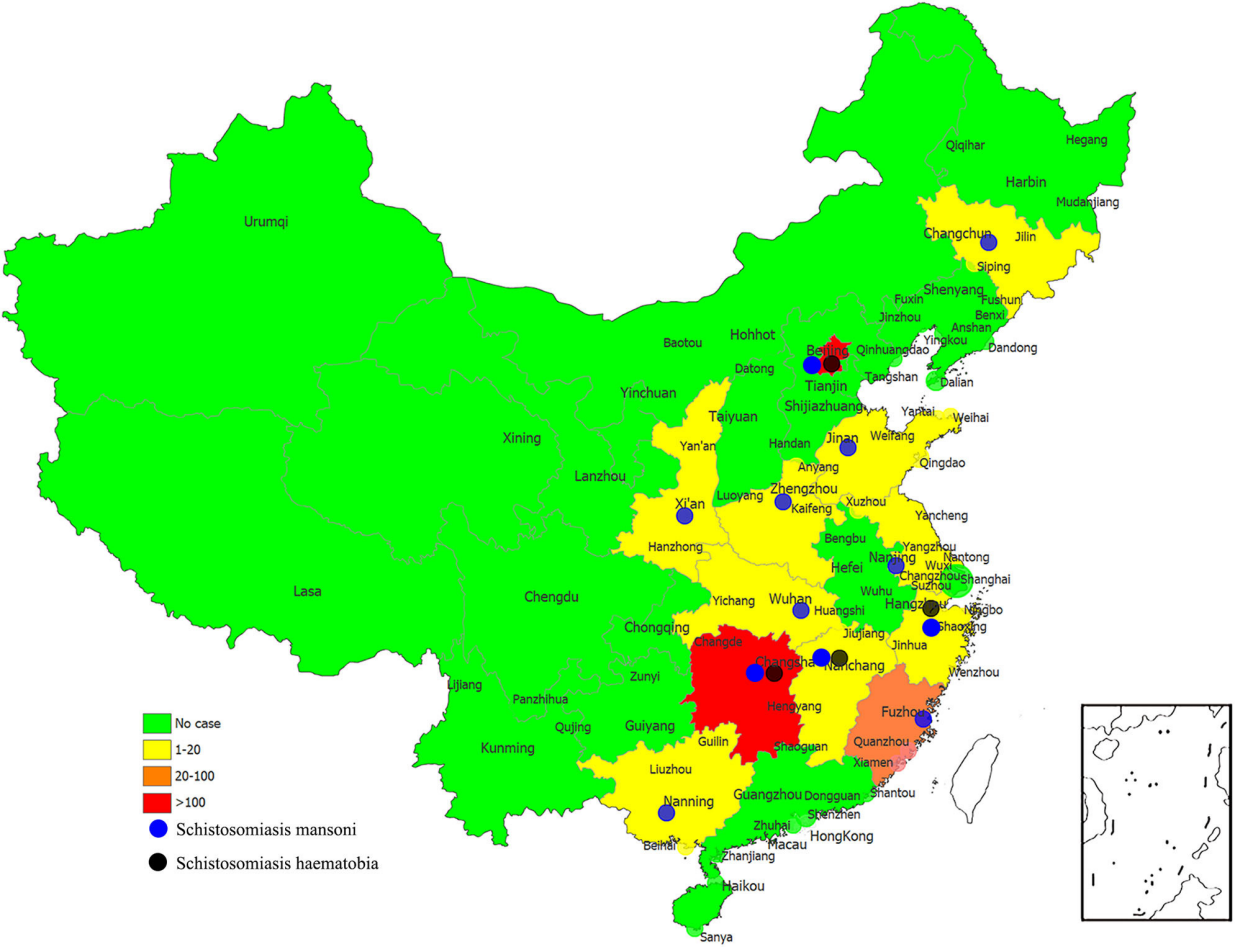
The numbers of imported cases of schistosomiasis mansoni and schistosomiasis haematobia in reported provinces of China.

Imported schistosomiasis in this study had been reported in 12 provinces/regions in China, with most cases found in Hunan (one case with *S. mansoni* infection and 184 cases with *S. haematobia* infection), Beijing (75 cases with *S. mansoni* infection and 39 cases with *S. haematobia* infection), and Fujian (23 cases with *S. haematobia* infection) provinces or municipalities (Figure 3). It has been noticed that those provinces and regions with a large number of migrant workers abroad usually have poor medical or public health infrastructure; therefore, with more cases misdiagnosed as other diseases or with the delayed diagnosis of *S. mansoni* or *S. haematobium* infections after returning to China, for example, one case in Hunan province got diagnosed as *S. haematobium* infection 84 months after he returned from Africa (23).

## Discussion

The importation of tropical diseases, including schistosomiasis, into China from endemic regions around the world is inevitable, unavoidable, and expected to be increased in the future due to the dramatically increased Chinese economic development and the boomed globalization. The fast-booming Chinese economy enormously increased trade with other countries and activities involved with international business. These activities include increased labor export from China, immigration, and tourism, all increasing the chance of importation of communicated diseases, from infection abroad, into China or the exportation of diseases infected in China to other countries. It was estimated that there were more than one million Chinese workers or tourists who visited or worked in Africa in 2013, and this number of travelers was ascended to three millions in 2019 (25, 34). In this study, we analyzed 355 cases of schistosomiasis imported from Africa from 1979 to 2019, 78 cases with schistosomiasis mansoni (21.97%), 262 cases with schistosomiasis haematobia (73.80%), and 15 cases with infection of unknown *Schistosoma* species (4.22%). Understanding the clinical manifestations and characteristics of imported schistosomiasis would greatly help Chinese physicians in making accurate diagnosis and appropriate treatment of these infections in China. A similar situation in Europe had 1,465 cases of imported schistosomiasis reported between 1997 and 2010, which was diagnosed by direct pathogen detection and serological antibody detection. Among them, 39% (570) of cases were determined as *S. mansoni* infection and 22% (318) of cases were infected with *S. haematobium* (35).

Schistosomiasis is known as the second most devastating parasitic disease, next to malaria, globally in terms of mortality, and more than 70 million disability-adjusted life years worldwide was lost (36). People get infected by skin contact with cercaria-contaminated freshwater where intermediate host snails exist. *S. japonicum, S. mansoni*, and *S. haematobium* are three major species that infect humans; however, only *S. japonicum* infection is endemic in some lake or marshland areas in China, while infections of *S. mansoni* and *S. haematobium* are widely endemic in southeastern Asia, Africa, and some areas in South America. Eventually, *Schistosoma* infections have serious adverse consequences on pregnancy and children's development and exacerbate social burden on the economies of households and governments, especially in poor countries such as in the sub-Saharan regions.

Human schistosomiasis has an acute infection stage and an advanced phase that show different clinical manifestations and disease development. Acute schistosomiasis was most common in travelers or migrants who had just visit schistosomiasis-endemic areas and have been exposed to water contaminated with schistosome cercariae or, for the first time, to people (usually children) living in endemic areas exposed to the parasite. Acute schistosomiasis occurs several weeks after the penetration of the schistosome cercariae through the skin, and the symptoms are related to the migration of the larvae within the human body. The typical clinical manifestations of acute schistosomiasis include a sudden onset of fever, fatigue, muscle pain, headache, abdominal pain, and eosinophilia that lasts for 2–10 weeks (37–40). If patients with acute schistosomiasis have not received proper anthelmintic chemotherapy timely, the disease may develop into a chronic form or progress rapidly where fibrosis, cirrhosis, and neoplasms might occur. Notably, some advanced pathological features could be developed, such as extended Glisson's sheath in the liver and portal hypertension, the latter of which could even happen during acute schistosomiasis mansoni (11).

In this study, some patients with schistosomiasis mansoni exhibited some acute-phase clinical manifestations such as fever (58.97%), diarrhea (55.12%), abdominal pain (54.41%), and increased eosinophil count (91.03%), which are different from those of an acute infection of local *S. japonicum* that is mainly referred to as Katayama fever, the prominent feature of the infection. Meanwhile, patients with schistosomiasis haematobia also had some acute-phase clinical manifestations, including fever (9.16%) and painless hematuria (53.82%). Noticeably, only about 50% of the patients were diagnosed as schistosomiasis at the acute phase. The misdiagnosis of schistosomiasis at an acute stage as other diseases may lead to the chance to be cured with anthelmintic therapy to be missed and could result in irreversible damage to tissue and health. Without timely treatment, people with *S*. *mansoni* infection may develop hepatic fibrosis and cirrhosis, and even esophageal varices complicated by ascites and hematemesis, which may rapidly result in death (41). *S. haematobium* infection causes urogenital schistosomiasis with a distinct symptom of hematuria with blood in the urine, often associated with frequent urination, painful micturition, and discomfort in the groin (42). The chronic infection may cause irreversible urinary tract damage and blockage, leading to obstructive uropathy, chronic bladder ulcers, and bladder carcinoma develop (43, 44). Furthermore, the chronic infection of *S. haematobium* in women causes infertility, preterm labor, anemia, menstrual disorders, and dyspareunia, a serious social stigma of poverty and disability in women in endemic areas (45, 46). To date, all imported cases of schistosomiasis haematobia in China were found in male patients because most of the migrant workers are men. The complications of schistosomiasis haematobia in women need to be further investigated in China.

Ectopic deposition of schistosome eggs may induce unexpected disorders. The central nervous system is the most common site for egg deposition, presenting as spinal cord compression or cerebropathy (46, 47). Cerebral schistosomiasis most often occurs during the acute period of *Schistosoma* infection, and the main clinical manifestations include meningoencephalitis symptoms such as fever, headache, vomiting, blurring of vision, sensory organ alteration, or Jacksonian seizure (46, 47). Spinal cord injury (more common in acute schistosomiasis) may manifest as acute transverse myelitis or sub-acute myeloradiculopathy and may cause paralysis or waist and leg pain complicated by muscle weakness, sensory loss, and urinary incontinence (46). However, these complications with *Schistosoma* egg deposition in the central nervous system have not been observed in these imported cases of *Schistosoma* infections in this study.

Due to the lack of knowledge for *S. mansoni* and *S. haematobium* infections in China, it was common to misdiagnose these infections as other diseases at the beginning. In this study, some imported schistosomiasis mansoni cases were misdiagnosed as eosinophilic gastroenteritis, ulcerative colitis, and malaria for up to 21–26 months without proper treatment, which deteriorated the patients' health as the disease developed (11). In addition, some cases of imported schistosomiasis with atypical clinical manifestations were even misdiagnosed as nephrotic syndrome with chronic *Salmonella* infection (7), nephrotic syndrome with secondary kidney amyloidosis (7), delayed paralysis (8), hyperreflexia (8), positive Babinski's sign (8), cauda-equina syndrome (9, 11), and myelopathy or placenta with schistosomal inflammatory granuloma in the perimedullary vein (11). Due to the diversity of egg deposition in different organs, schistosomiasis haematobia may appear as diverse clinical manifestations at the chronic stage, including urinary, reproductive, digestive, respiratory, circulatory, and nervous organ system disorders. Some only reveal dermatological manifestations. Most cases of imported schistosomiasis haematobia were initially misdiagnosed as prostatitis (16, 17, 24, 29), urinary tract infection (16, 20, 22, 26, 31), renal tuberculosis (16, 19, 32), or bladder tumors (12, 30, 32) prior to the definitive diagnosis. However, some other common misdiagnoses of schistosomiasis haematobia such as urinary tract stone, acute appendicitis, chronic appendicitis, enterospasm, and gastrointestinal dysfunction were not reported in these imported schistosomiasis haematobia cases in this study.

The definitive diagnosis of *Schistosoma* spp. infection relies on the identification of egg(s) in urine, stool, or biopsy specimens. In this analysis, the identification of *S. mansoni* eggs in stool samples or miracidia hatched from stool samples had occurred in 88.46% (69/78) of cases. Interestingly, it has been demonstrated that the characteristic mucosal granuloma and damage caused by *Schistosoma* infection can be identified by colonoscopy or cystoscopy. The biopsy of rectal and bladder tissues under endoscopy is of great significance and assistance in the diagnosis of intestinal and urogenital schistosomiasis in the absence of *Schistosoma* eggs found in fecal and urine samples. In this analysis, the *S. mansoni* eggs were identified in 14.10% (80/262) of cases in rectal mucosal biopsy by colonoscopy, and *S. haematobium* eggs were identified in 4.20% (11/262) of cases with cystoscopy plus biopsy. There were two cases of S. *haematobium* infection with the egg identified by rectal biopsy (0.76%, 2/262). Since the current standard procedure is not able to detect eggs in all infected patients, the final diagnosis of schistosomiasis depends on the full consideration of multiple criteria such as clinical manifestation, explosion in the endemic area, egg detection in urine and/or fecal sample, pathological examination by endoscopy, and serological measurement of specific antibodies or antigens (12, 40).

Even though the detection of schistosome-specific antigens has been proven to be beneficial for the diagnosis of current *Schistosoma* infections, there is only the anti-*S. japonicum* antibody assay kit commercially available in China. In this analysis, 189 out of 262 cases of imported schistosomiasis haematobia showed serologically positive results to *S. japanicum* extract antigens by IHA or ELISA assay. The results indicate that the *S. japanicum* antibody detection kit can be used as an alternative tool to screen for infections of *S. mansoni* or *S. haematobium* imported from other countries. However, the antibody cross-positive rate was only 72.13% in cases infected with *S. haematobium*. It is necessary to develop species-specific immunodiagnostic or molecular tools to detect infections of *S. mansoni* and *S. haematobium* with high priority (48), especially for differentiating between active infections and previous exposure (49). *Schistosoma* real-time PCR was used to detect infections in samples of urine and stool, and it was found to have significantly increased sensitivity and specificity compared to conventional microscopic examination (38, 39, 49).

As an effective anti-schistosomal medicine, praziquantel was used to treat all diagnosed cases of imported schistosomiasis at a single dose of 40 mg/kg (39). This dose was well tolerated by all patients without an apparent adverse effect. The main adverse reactions that we observed included abdominal pain, diarrhea, decreased appetite, transient drowsiness, and fever within 24 h of drug administration as described (50). It has been reported that the combinations of artesunate–praziquantel or artesunate–mefloquine achieve satisfactory cure rates and egg burden reduction rates for the treatment of *S. japonicum* infection (50, 51). However, artemisinin seems to fail to achieve a satisfactory efficacy for the treatment of *S. haematobium* infection (52, 53). Although praziquantel has been extensively used across the world, fortunately, there is no direct evidence of resistance to praziquantel in *Schistosoma* spp. No drug-resistant isolate has been identified, even though praziquantel-resistant *S. mansoni* was observed in the laboratory (54). Nevertheless, there is still a risk of emergence of drug resistance due to extensive monotherapy (54).

The risk for the transmission of imported schistosomiasis in local population is present if there is an appropriate snail vector existing in the area. In 2013, more than 120 locals or tourists were identified to be infected with *S. haematobium* on Corsica Island of France, a non-endemic region for schistosomiasis (55, 56). DNA sequencing analysis of the parasite confirmed that the origin of the identified *S. haematobium* was Senegal in West Africa (57). It is apparent that the transmission of *S. haematobium* had occurred on the island possibly due to the importation of *Schistosoma* parasite from Senegal in Africa and the existence of an appropriate snail host on the island. The snail *Biomphalaria straminea*, the intermediate host of *S. mansoni*, has been identified in Hongkong since 1974 and in Shenzhen since 1981 (58, 59). The existence of *B. straminea* was even observed in Dongguan and Huizhou regions in Guangdong Province (6, 60). The existence of intermediate host *B. straminea* and the imported cases of *S. mansoni* infection could cause the spread of infection and make such endemic in the area. The potential transmission of *S. mansoni* infection in the regions raises a big concern, and local public health authority has paid serious attention to prevent the potential spread of the infection (6). More investigations should be carried out to determine detailed information on snail distribution and ecology, vulnerability to *S. mansoni* miracidial infection, and the potential to transmit the infection of *S. mansoni* to people. So far, the *S. haematobium* intermediate host *Bulinus* snail has not been found in China; however, there is still a possibility for such to be imported from abroad *via* air or maritime transportation as international trade and travel is increased. Thus, management and surveillance of international travel and tourists from endemic areas should be strengthened. Due to the increased imported cases of schistosomiasis and the existence of the potential intermediate host snail, it is urgently needed to evaluate the transmission risk of schistosomiasis mansoni/haematobium in China as well as to develop serological screening methods and diagnostic techniques (39, 61).

## Data Availability Statement

All datasets generated for this study are included in the article/supplementary material.

## Ethics Statement

The data were collected from peer-reviewed published literature and public database with approved ethics statement or patients' consent, without exposure of patients' identity information.

## Author Contributions

YZ conceived the study. LW and XW performed data collection and curation. XL performed formal analysis. XZ and FW performed data investigation. ZQ and MH performed the statistical analysis. LW and XW wrote the draft of the manuscript, which was revised by LW and YZ. All authors contributed to the article and approved the submitted version.

## Conflict of Interest

The authors declare that the research was conducted in the absence of any commercial or financial relationships that could be construed as a potential conflict of interest.
